# High power Na_3_V_2_(PO_4_)_3_ symmetric full cell for sodium-ion batteries†

**DOI:** 10.1039/d0na00729c

**Published:** 2020-10-20

**Authors:** Milan K. Sadan, Anupriya K. Haridas, Huihun Kim, Changhyeon Kim, Gyu-Bong Cho, Kwon-Koo Cho, Jou-Hyeon Ahn, Hyo-Jun Ahn

**Affiliations:** Department of Materials Engineering and Convergence Technology, Gyeongsang National University 501 Jinju-daero Jinju Gyeongnam 52828 South Korea ahj@gnu.ac.kr jhahn@gnu.ac.kr

## Abstract

Sodium-ion batteries (SIBs) are a viable substitute for lithium-ion batteries due to the low cost and wide availability of sodium. However, practical applications require the development of fast charging sodium-ion-based full-cells with high power densities. Na_3_V_2_(PO_4_)_3_ (NVP) is a bipolar material with excellent characteristics as both a cathode and an anode material in SIBs. Designing symmetric cells with NVP results in a single voltage plateau with significant specific capacity which is ideal for a full cell. Here we demonstrate for the first time a tremendous improvement in the performance of NVP symmetric full cells by introducing an ether-based electrolyte which favors fast reaction kinetics. In a symmetric full cell configuration, 75.5% of the initial capacity was retained even after 4000 cycles at 2 A g^−1^, revealing ultra-long cyclability. Excellent rate performances were obtained at current densities as high as 1000C, based on the cathode mass, revealing ultrafast Na^+^ transfer. The power density obtained for this NVP symmetric cell (48 250 W kg^−1^) is the best among those of all the sodium-ion-based full cells reported to date.

## Abstract 2

The study details the electrochemical characterization of NVP anodes and symmetric NVP cells in a DME electrolyte for the first time.

## Graphical abstract

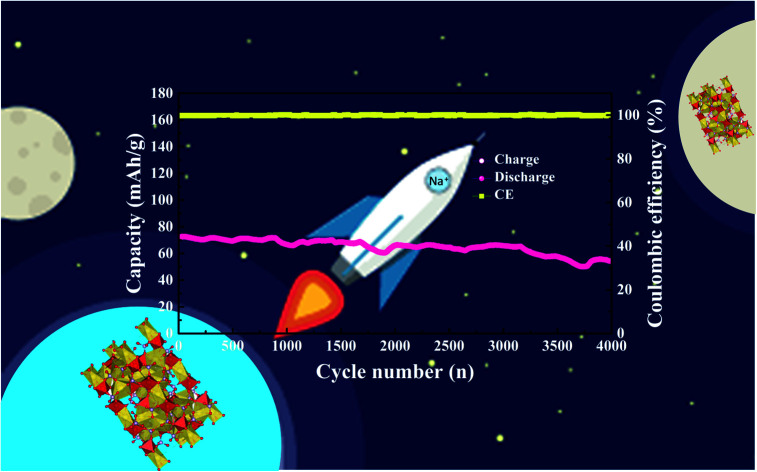

## Introduction

The development of energy storage devices is important, due to the prevalence of portable electronics and electric vehicles. Lithium-ion batteries (LIBs) are currently exploited as typical energy storage devices. However, the scarcity of lithium resources in the earth's crust is a concern that needs to be addressed.^1,2^ Sodium-ion batteries (SIBs) are considered an appropriate candidate that can replace the present LIB technology. The wide availability and low cost of sodium resources, along with the fact that Na and Li have analogous electrochemical characteristics, have made SIBs tremendously attractive.^3,4^ However, the practical application of SIBs requires the development of sodium-ion based full cells which are on par with the current LIB technology.^5,6^ However, full cells consisting of alloying and conversion materials experience capacity decay and exhibit poor performances due to the volume expansion and pulverization of active materials.^7^ In contrast, full cells with insertion materials do not experience significant volume changes during sodiation and desodiation processes. Moreover, insertion materials possess a stable structure, which results in faster reaction kinetics compared to conversion and alloying materials.^8^ Hard carbon which is a popular anode does not exhibit good rate performances. Hence, insertion materials like NASICON-type (sodium super ionic conductor) compounds are widely studied, as they possess many vacant spaces for Na^+^ ion diffusion, promoting fast kinetics.^9^

Sodium vanadium phosphate (Na_3_V_2_(PO_4_)_3_, NVP) is a typical NASICON structured material. With a discharge voltage of ∼3.4 V, corresponding to the V^3+^/^4+^ redox reaction,^10^ NVP is considered to be a typical SIB cathode material. The additional low voltage plateau at ∼1.6 V, denoting the V^3+^/^2+^ redox reaction, affords NVP the ability to act as an anode material as well.^11^ Thus, the construction of symmetric cells with NVP can provide a single voltage plateau at 1.8 V with significant specific capacity. Most of the previous reports on symmetric full cells have focused only on the modification of NVP cathodes.^12–15^ Even though the NVP cathode possesses superior characteristics in terms of the structural stability and reaction mechanism, the material's low electronic conductivity limits its electrochemical performance.^10^ Previous studies have tried to overcome this drawback by adopting doping strategies and utilizing conductive carbon matrices. For instance, Matsumoto *et al.* reported an NVP embedded carbon nanofiber composite.^12^ In an expensive ionic liquid electrolyte system, the symmetric full-cell delivered a capacity of 48 mA h g^−1^ at a current density of 100C with good cycling performance up to 3000 cycles retaining 54 mA h g^−1^. Other complex electrode modification strategies attempting to address NVP's low electronic conductivity have resulted in comparatively low electrochemical performance.^16–18^ A recent NVP-based full cell in an asymmetric configuration with Bi and Sn anodes showed high reversible capacity in a DEGDME-based electrolyte;^19–21^ however, its rate and cycling performances were not adequate. In contrast, our group recently reported the excellent rate performance and long-term cycling performance of an NVP cathode in a dimethoxyethane (DME) electrolyte.^22^ Following this trail, here we report the electrochemical properties of NVP as an SIB anode utilizing the low voltage plateau and extend its application to symmetric full cells based on NVP, employing a DME electrolyte for the first time.

## Results and discussion

**Fig. 1:**
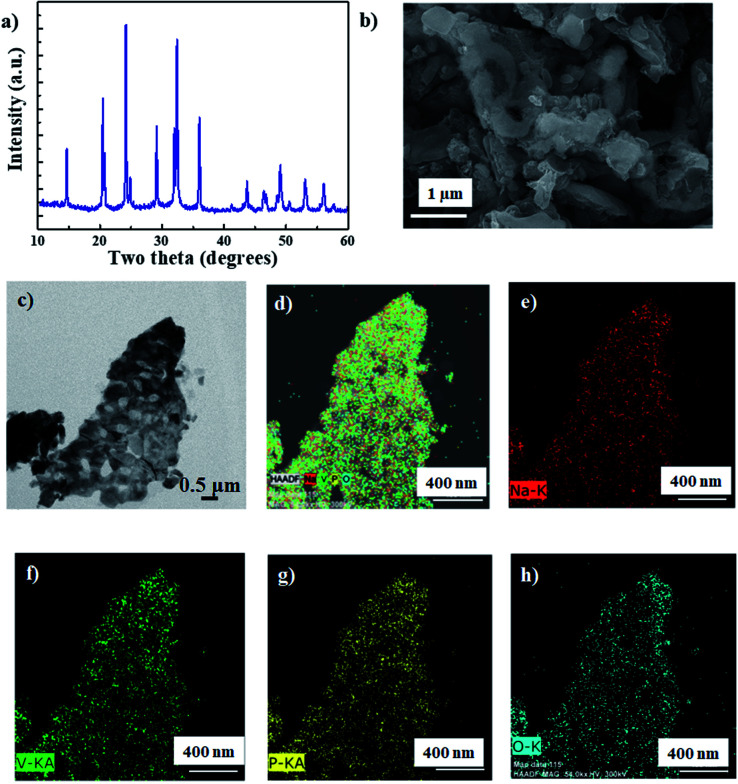
(a) X-ray diffraction (XRD) spectrum, (b) field emission scanning electron microscopy (FESEM) image, and (c) transmission electron microscopy (TEM) image of NVP nanoparticles. (d–h) Energy dispersive X-ray spectroscopy (EDS) map of elements in NVP nanoparticles.

NVP was synthesized using a sol–gel method, as per our previous report.^22^Fig. 1a shows the X-ray diffraction (XRD) spectrum of the synthesized NVP nanoparticles. All the peaks can be indexed to the rhombohedral NVP phase without any impurity.^23^ The morphology of NVP was analyzed using a combination of field emission scanning electron microscopy (FESEM) and transmission electron microscopy (TEM), the results of which are depicted in Fig. 1b and c, respectively. These images demonstrate that the synthesized NVP particles are highly porous, with an average size in the order of nanometers. The results of energy dispersive X-ray spectroscopy (EDS) mapping (Fig. 1d–h) show that the constituent elements (Na, V, P, and O) are uniformly distributed throughout the particle.

**Fig. 2:**
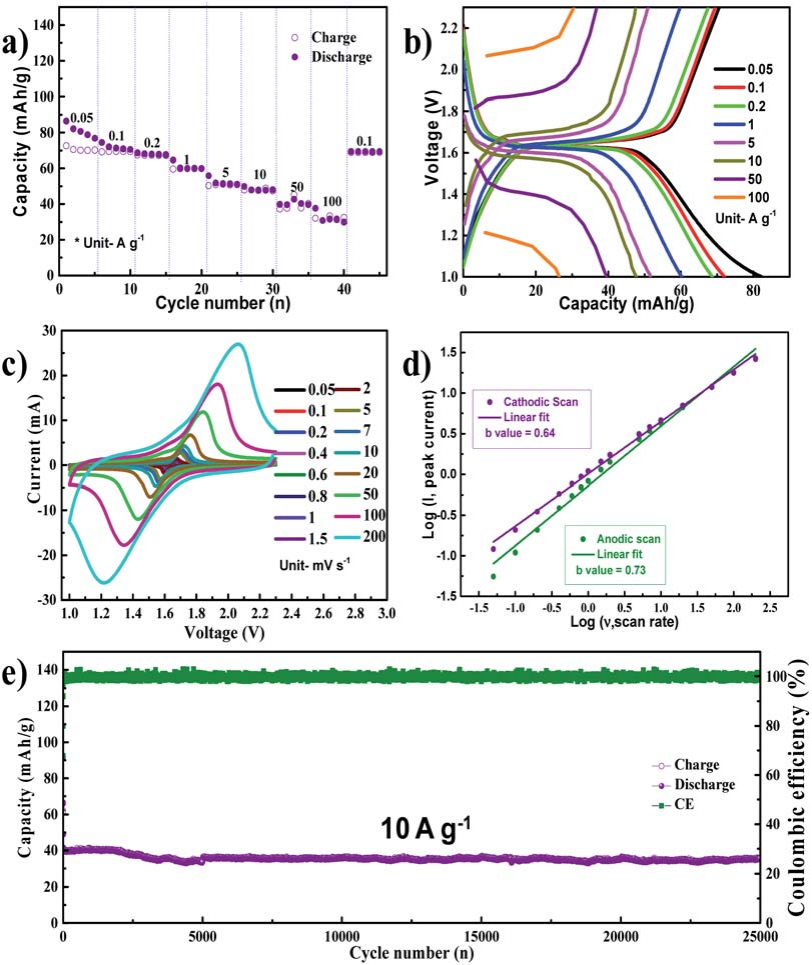
(a) Results of rate performance tests conducted on the NVP anode and (b) the corresponding voltage profile during testing. (c) Results of cyclic voltammetry (CV) tests conducted at scan rates varying from 0.05 mV s^−1^ to 200 mV s^−1^. (d) Logarithmic relationship between peak current (*I*) and scan rate (*ν*). (e) Results of long-term cycling performance tests conducted at a current density of 10 A g^−1^.

The cyclic voltammetry (CV) curves of the Na/NVP half-cell obtained at a scan rate of 0.05 mV s^−1^ are shown in Fig. S1a.† Single anodic and cathodic peaks were observed around 1.6 V. The NVP anode exhibited a low overpotential of 0.026 V, with similar peak intensities and positions noted in all subsequent cycles. A representative voltage profile (measured at a current density of 100 mA g^−1^) is shown in Fig. S1b,† while the corresponding cycle performance is shown in Fig. S1c.† The voltage profile reveals a single voltage plateau around 1.6 V, which is analogous with the CV curves. After the initial cycle, the coulombic efficiency of the anode increased from 84% to 100%. The half-cell shows excellent rate performance (Fig. 2a) upon sequentially increasing the current densities from 0.05 A g^−1^ to 100 A g^−1^. The Na/NVP half-cell delivered capacities of 65, 62, 60, 53, 45, 43, 33, and 30 mA h g^−1^ at the respective current densities from 0.05 A g^−1^ to 100 A g^−1^. Following this, a capacity of 62 mA h g^−1^ was restored when the current density was reduced to 0.1 A g^−1^. Fig. 2b depicts the voltage profiles during the rate performance. It is worth noting that a single plateau was observed in all voltage profiles, irrespective of the current density.

To understand the kinetics of the NVP anode, CV was conducted at scan rates ranging from 0.05 mV s^−1^ to 200 mV s^−1^ (Fig. 2c). The charge storage kinetics was elucidated by plotting the logarithm of the peak current with respect to the logarithm of the scan rate, as shown in Fig. 2d, with the slope of this linear plot (*b* value) dictating the form of charge storage kinetics in the electrode.^24^ The *b* value was 0.73 for anodic scans and 0.64 for cathodic scans. These values indicate that a mixed reaction mechanism involving diffusive and capacitive phenomena occurs during both the anodic and cathodic scans, which is a possible reason for the excellent high rate performance. The long term cycling performance of the NVP anode is shown in Fig. 2e at a current density of 10 A g^−1^. The NVP anode shows a stable long-term cycling performance, delivering an initial capacity of 40 mA h g^−1^ with a capacity retention of 89% after 25 000 cycles, which corresponds to a very low capacity decay of 0.00044% per cycle. The selected voltage profile of the NVP anode during long-term cycling is shown in Fig. S2a.† While cycling, it can be seen that the overpotential of the NVP anode is found to be insignificant. Following these long-term tests, five additional charge–discharge cycles were performed at a current density of 100 mA g^−1^, and the corresponding voltage profiles are shown in Fig. S2b.† The voltage profiles match exactly with that in Fig. S1b,† indicating that the performance of the electrode remains stable even after long-term cycling. This is supported by the CV scan conducted after long-term testing (at 0.1 mV s^−1^). The CV curves (Fig. S2c†) obtained are similar to the CV scan of the pristine electrode shown in Fig. S1.† The FESEM image of the electrode after 25 000 cycles is shown in Fig. S2d,† indicating the absence of pulverization and volume expansion in the NVP nanoparticles even after prolonged cycling. Electrochemical impedance of the electrode before cycling and after the 25 000^th^ charge–discharge cycle is shown in Fig. S3.† The pristine NVP anode shows a charge transfer resistance (*R*_ct_) of 13 Ω, which increased to 16 Ω after 25 000 cycles. The GITT profile of the NVP anode is shown in Fig. S4,† where ohmic drops of 0.4 mV and 0.6 mV can be observed during sodiation and desodiation, respectively. The low *R*_ct_, overpotential, and mixed capacitive characteristics of the NVP anode contribute to the excellent high-rate performance.

**Fig. 3:**
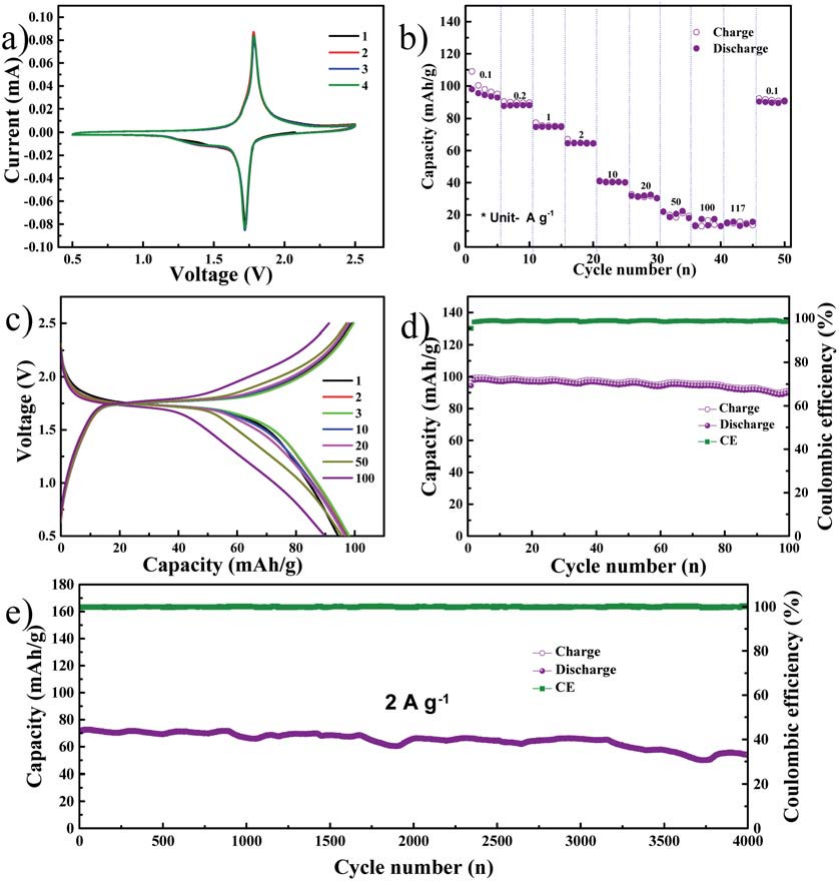
(a) Results of CV tests conducted on the symmetric NVP cell in the DME electrolyte at a scan rate of 0.1 mV s^−1^ in the 0.5 V to 2.5 V voltage window. (b) Results of rate performance tests conducted on the symmetric cell. (c) Voltage profile and (d) the corresponding cycling performance at a current density of 0.1 A g^−1^. (e) Cycling performance at a current density of 2 A g^−1^.

The previous report from our group detailed the excellent electrochemical performance of NVP as an SIB cathode material using the DME electrolyte.^22^ Hence, we assembled a symmetric full cell consisting of the NVP anode optimized in the present study and the NVP cathode optimized in our previous work. The CV profile of the symmetric NVP cell, obtained at a scan rate of 0.1 mV s^−1^, is shown in Fig. 3a. A single anodic peak at 1.78 V and a cathodic peak at 1.72 V were observed which is exactly similar to previous reports.^28,33^ The peak positions and intensities are identical in all four cycles, indicating that the electrochemical reactions in the full cell are highly reversible. Fig. 3b shows the rate performance of the NVP symmetric cell, with current densities ranging from 0.1 A g^−1^ (0.85C) to 117 A g^−1^ (1000C), based on the mass of the cathode. The symmetric cell exhibited capacities of 95, 88, 74, 64, 41, 31, 20, 17, and 15 mA h g^−1^ at current densities of 0.1, 0.2, 1, 2, 10, 20, 50, 100, and 117 A g^−1^, respectively. A capacity of 91 mA h g^−1^ was retained when the current density was finally reduced to 0.1 A g^−1^. The voltage profile during the rate performance test is provided in Fig. S5a.† It can be noted that the full cell exhibits a single voltage plateau with low overpotential at all current densities. Further, the NVP symmetric cell was cycled at a low current density of 100 mA g^−1^ for 100 charge–discharge cycles: Fig. 3c and d show the selected voltage profiles and cycling performance, respectively. In the former image, a single plateau can be observed at 1.74 V, while the latter image details that the initial reversible capacity of the full cell is 99 mA h g^−1^ with a coulombic efficiency of 95%. After 100 cycles, a capacity of 91 mA h g^−1^ was retained, while the coulombic efficiency was almost 100%. Fig. 3e shows the long-term cycle performance of the full cell, tested at a current density of 2 A g^−1^ (17C based on the cathode), and the corresponding voltage profiles are shown in Fig. S5c.† The NVP symmetric full cell shows stable long-term performance, retaining 75.5% of its initial capacity (71.7 mA h g^−1^). This result is far better than the best among previous reports, such as the 60% capacity retention reported by Matsumoto *et al.* following 3000 cycles in an expensive ionic liquid-based electrolyte.^12^ The full cell's electrochemical impedance after long-term cycling is shown in Fig. S5b.† The *R*_ct_ is extremely low, at 13 Ω, which indicates that the cell retains excellent kinetics even after 4000 cycles. Fig. S5d and e† show FESEM images of the NVP anode and cathode, respectively, after 4000 cycles. The electrodes displayed no signs of pulverization or volume expansion, which can be the reason for the stable long-term cycle performance of the full cell. CV scans of the full cell at different rates (varying from 0.1 mV s^−1^ to 50 mV s^−1^) are shown in Fig. S6a.† At all scan rates, single anodic/cathodic peaks can be observed with a slight overpotential. As before, the *b* value is evaluated from the ratio of the logarithm of the scan rate to the logarithm of the peak current and is shown in Fig. S6b.† During the anodic and cathodic scans, the *b* values are 0.58 and 0.56, respectively, indicating that the observed capacity originates from the diffusion mechanism. Fig. S7† shows the GITT profile of the NVP symmetric cell. Here, an ohmic drop of 0.004 V was observed during discharge, while a similar drop of 0.005 V was noted during charge. These low ohmic drops indicate low heat dissipation during charging or fast discharging.^25^ Additionally, these values are low compared to the present commercial batteries, indicating the prospects of safe charging and discharging at high current rates with NVP symmetric cells.^26^

The electrochemical performance of the NVP symmetric cell in 1 M NaClO_4_ in EC/PC with a 5% FEC electrolyte is shown in Fig. S8.† The rate performance at different current densities and the corresponding voltage profiles are summarized in Fig. S8a and b,† respectively. At low current densities the cell capacities were comparable to that in the ether electrolyte; however at high current densities, low capacities were obtained. Additionally, from the voltage profile in Fig. S8b,† it can be noted that the overpotential in the carbonate electrolyte is higher, while the plateau capacity is lower than that in the case of the ether electrolyte. The unstable, thick cathode interface component in the carbonate electrolyte^22^ may have resulted in the poor rate performance of the symmetric cell. Fig. S8c and d† show the voltage profile of the NVP symmetric cell in the carbonate electrolyte at a current density of 2 A g^−1^ and its corresponding cycling performance. Capacity retention with the carbonate electrolyte was poor compared to that with the ether electrolyte. A comparison of the performance of the NVP symmetric cell in the present report with those in previous reports on NVP-based symmetric cells is summarized in Table S1,† highlighting that the performance improvement with our modifications is more effective. Similarly, comparisons between the rate and cycling performances of previously reported NVP symmetric cells and those detailed in this report are shown in Fig. S9a and b,† respectively. In terms of the cycling performance and rate performance, our full cell outperforms all the previously reported full cells in a symmetric configuration.^13–16,18,27–34^ This can be attributed to the excellent kinetics offered by the DME electrolyte in the NVP cathode and NVP anode. A Ragone plot comparing the symmetric NVP cell with different energy storage devices is shown in Fig. S10.† It should be noted that the NVP-symmetric full cell exhibits a power density higher than that of batteries and an energy density higher than that of supercapacitors. In addition, our NVP symmetric cell exhibited higher power density than the best reports of full cells based on alloying materials such as Bi and Sn,^19–21^ delivering a maximum power density of 48 250 W kg^−1^ (based on the total weight of the anode and the cathode) at a current density of 117 A g^−1^. This value is the highest among the power densities of all the previously reported full cells and brings NVP-based symmetric cells one step closer to commercialization.^5,6^ This covetable achievement can be attributed to the synergistic effects of the 3D diffusion pathways in NVP, that facilitate the excellent Na^+^ transfer rate, and the utilization of an ether-based electrolyte in this work, which favors fast reaction kinetics.

## Conclusions

In summary, this study investigates the electrochemical properties of NVP nanoparticles prepared using a simple sol–gel method as an anode and extends its applicability to a symmetric NVP-based full cell utilizing a DME electrolyte. The NVP anode exhibited excellent rate capability and long-term cycling performance, delivering a capacity of 30 mA h g^−1^ at an extremely high current density of 100 A g^−1^. Moreover, the NVP anode retained 89% of its initial capacity (40 mA h g^−1^) after 25 000 cycles at 10 A g^−1^, with a capacity degradation rate of 0.00044% per cycle. The NVP symmetric cell (full cell) demonstrated good rate performance even at a high current density of 50 A g^−1^, delivering a capacity of 20 mA h g^−1^, and long-term stability up to 4000 cycles, retaining 75.5% of its initial capacity of 71.7 mA h g^−1^. Based on the total weight of the anode and cathode, the symmetric cell delivered a maximum power density of 48 250 W kg^−1^, which is the highest among all the previous reports on sodium-ion-based full cells.

## Conflicts of interest

There are no conflicts to declare.

## Supplementary Material

NA-002-D0NA00729C-s001
