# Supplementary material for: High power Na3V2(PO4)3 symmetric full cell for sodium-ion batteries
Source: Nanoscale Adv. 2020 Oct 20;2(11):5166–70. doi: 10.1039/d0na00729c (PMC9419746; doi:10.1039/d0na00729c)
Supplement: NA-002-D0NA00729C-s001 [file NA-002-D0NA00729C-s001.pdf]

Electronic Supplementary Material (ESI) for Nanoscale Advances.
This journal is © The Royal Society of Chemistry 2020

Electronic Supplementary Information

High power $Na_3V_2(PO_4)_3$ symmetric full cell for sodium-ion batteries

Milan K. Sadan, Anupriya K. Haridas, Huihun Kim, Changhyeon Kim, Gyu-Bong Cho, Kwon-Koo Cho, Jou-Hyeon Ahn*, Hyo-Jun Ahn*

Experimental

Materials and methods

Sodium vanadium phosphate (NVP) was synthesized using a sol-gel method, as per our previous report.[1] Sodium hydroxide (NaOH, 98%, Alfa Aesar, Haverhill, Massachusetts, USA), ammonium dihydrogen phosphate ($NH_4H_2PO_4$, 99%, Acros Organics, Geel, Belgium), ammonium vanadium oxide (NH_4VO_3, 99%, Alfa Aesar), and citric acid ($C_6H_8O_7$, 98%, Sigma Aldrich, St. Louis, Missouri, United States) precursors were dissolved in deionized (DI) water, and mixed using a magnetic stirrer. The aqueous precursor solution was converted to a gel by raising the temperature to 120 °C. This gel was subsequently pretreated at 300 °C for 4 h in nitrogen atmosphere, following which, the temperature was raised to 800 °C at a ramp of 5 °C min^{-1}, where it was maintained for 7 h. The powder obtained after this process was washed several times with ethanol and DI water and stored in the oven overnight.

Material and electrochemical characterization

For characterization of the fabricated NVP material, crystallographic structures were analyzed with X-ray diffractometry (XRD), using the Bruker D8 (Bruker Corporation, Billerica, Massachusetts, USA). The microscopic morphology was observed using a combination of field-emission scanning electronic microscopy (FESEM) and transmission electron microscopy (TEM), using the TESCAN MIRA3 (LM, Brno, Czechia) and JEOL 2010 FEG (JEOL Ltd., Tokyo, Japan) tools, respectively.

For electrochemical measurements, Swagelok-type cells were fabricated with Na metal as the counter electrode. The electrode was prepared by mixing NVP, Ketjenblack, and polyvinylidene fluoride in an 8:1:1 ratio with N-methyl-2-pyrrolidone. The slurry was cast on carbon-coated aluminum foil with an approximate loading of 1 mg cm^{-2}. The sodium hexafluorophosphate (1 M $NaPF_6$) in ethylene glycol dimethyl ether (DME) electrolyte was prepared in-house. Glass fiber filter paper (GF/D, Whatman PLC, Maidstone, UK) and Celgard 2400 were coupled together, for use as separators. The cells were assembled in an argon-filled glove box and tested at room temperature. Cell cycling was performed using a galvanostat (WBCS 3000L, WonA Tech Co. South Korea), while cyclic voltammetry and electrochemical impedance spectroscopy were performed using a VMP3 multi-channel potentiostat (Biologic, Seyssinet-Pariset, France). The full cell was assembled using an NVP cathode with a mass ratio of 1:2.5. Before assembly, this electrode was pre-cycled twice, to overcome the irreversible capacity.

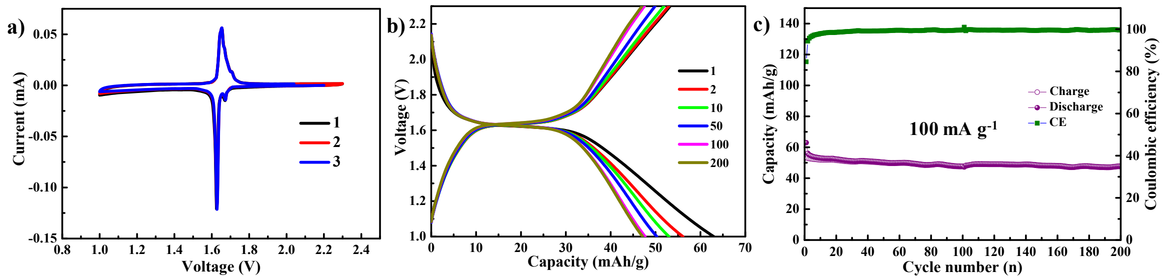

Figure S1. a) Cyclic voltammetry (CV) tests conducted on the NVP anode at a scan rate of 0.05 mV s^{-1} in the 1 V to 2.3 V voltage window. b) Voltage profile, and c) corresponding cycling performance at a current density of 100 mA g^{-1}.

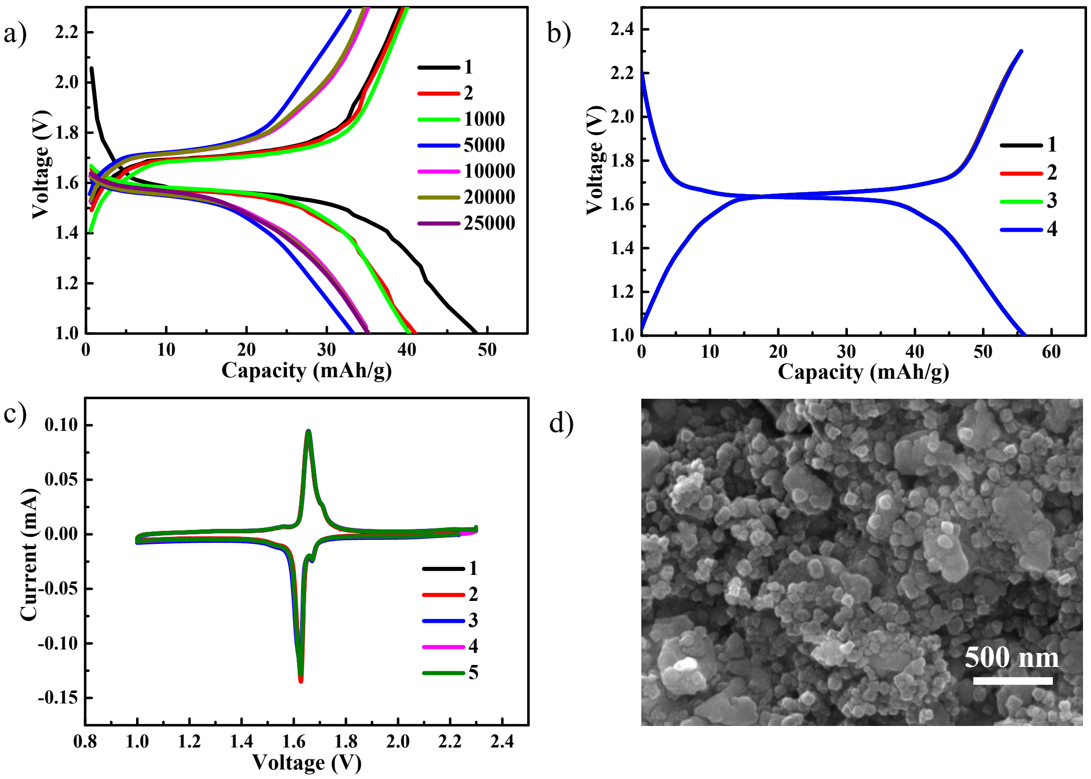

Figure S2. a) Voltage profile of NVP anode while cycle performance tests. b) Voltage profile of NVP anode after long-term cycle testing at a current density of 100 mA g^{-1}. c) CV test conducted at a scan rate of 0.1 mV s^{-1} after long-term cycle testing. d) FESEM image of NVP anode after long-term cycle testing.

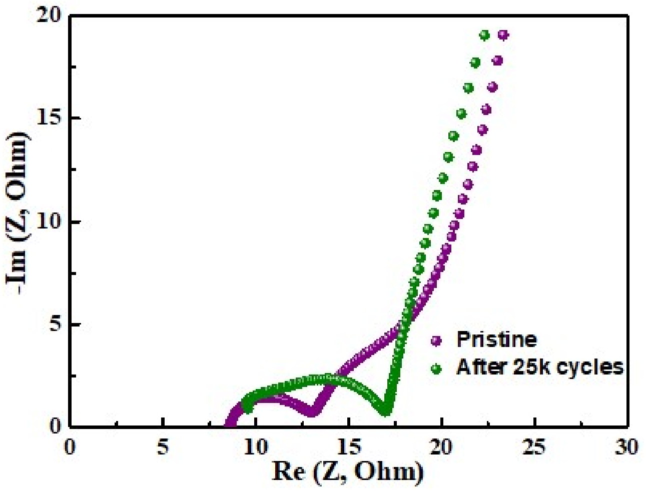

Figure S3. Nyquist plot of the NVP anode before and after long-term cycle performance testing.

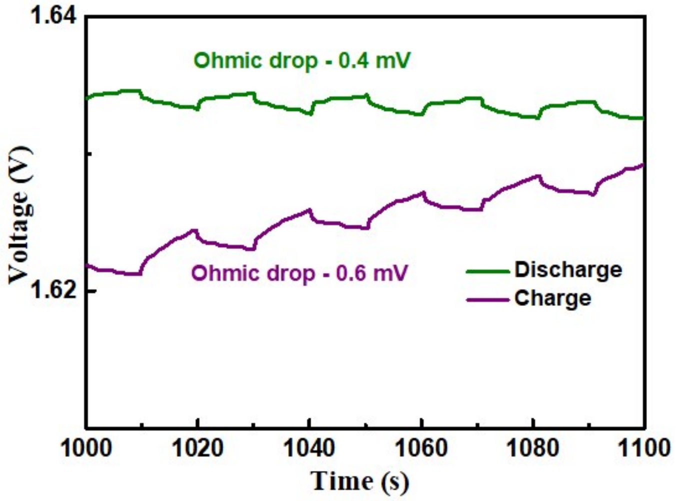

Figure S4. Galvanostatic Intermittent Titration Technique (GITT) profile of the NVP anode.

The diffusion coefficient of the NVP anode is calculated from GITT profile as $4.38\times10^{-9}cm^2\ sec^{-1}$.

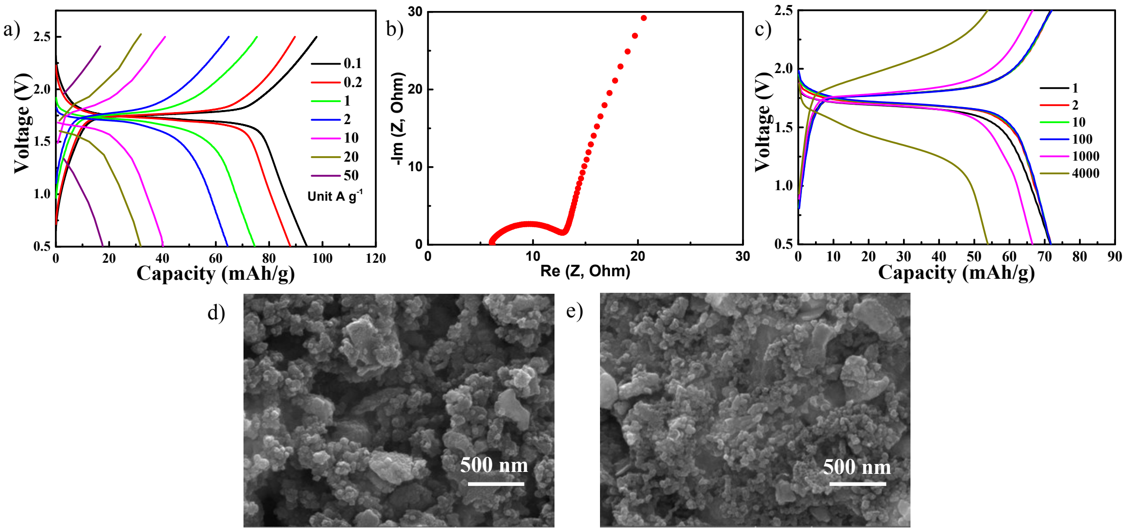

Figure S5. a) Voltage profile of the symmetric NVP cell in DME at different current densities. b) Nyquist plot of the full cell. c) Voltage profile of the full cell during cycling tests conducted at a current density of 2 A g^{-1}. FESEM image of the d) anode and e) cathode of the symmetric cell after 4000 cycles.

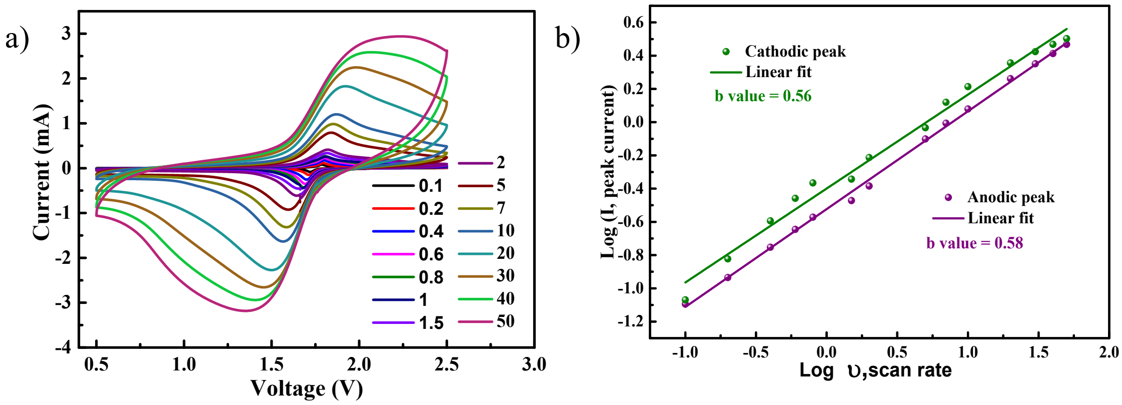

Figure S6. a) CV tests conducted on NVP cell in DME at scan rates varying from 0.1 mV s^{-1} to 50 mV s^{-1}.

b) Logarithmic relationship between peak current (I) and scan rate (ν).

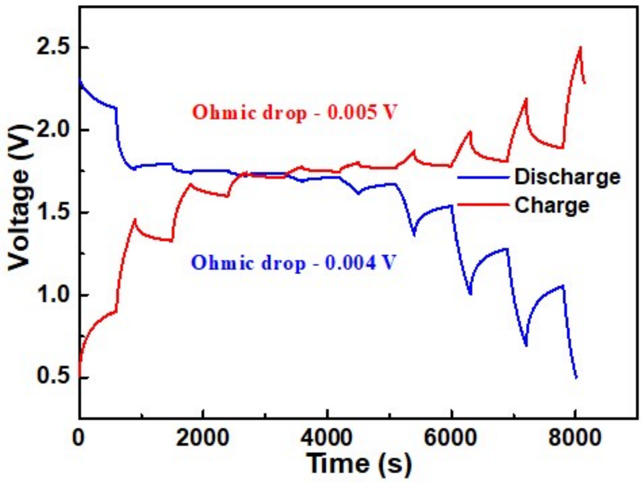

Figure S7. GITT profile of the symmetric NVP cell.

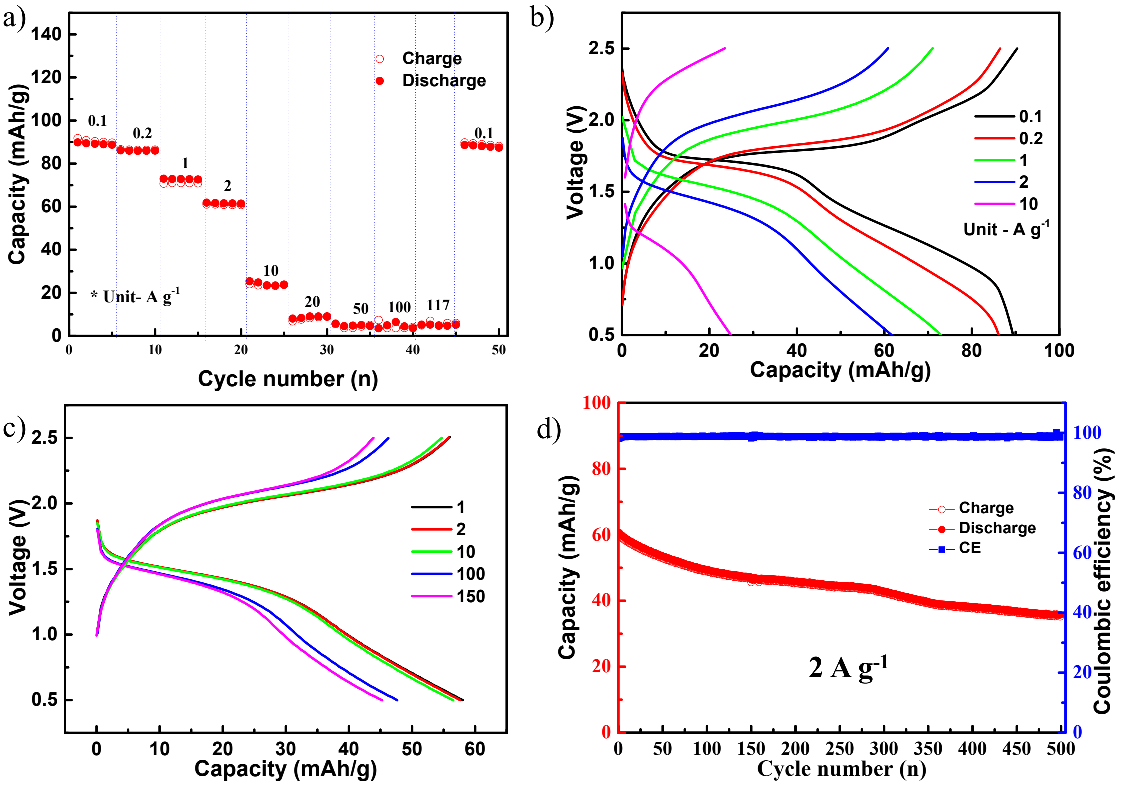

Figure S8. a) Rate performance tests conducted on the symmetric NVP cell in carbonate electrolyte, and b) corresponding voltage profile during testing. c) Voltage profile, and d) corresponding cycling performance at a current density of 2 A g^{-1}.

Table S1. Summary of characteristics of NVP cells reported in the literature.

**Sl. No.**	**Electrode**	**Electrolyte**	**Voltage window**	**Reversible capacity**	**Initial coulombic efficiency**	**Cycling performance**	**Rate performance**	**Mass ratio of Anode : Cathode**		**Reference Mass**	**Ref**
1	NVP@C core–shell	1 M $NaClO_4$ in PC	1 V–2.2 V	90.9 mAh g^{-1} (2 C)	–	73.6 mAh g^{-1} (50 cycles)	–	5	3	based on cathode	2
2	NVP:rGO-CNT	1 M $NaClO_4$ in PC + 5% FEC	1 V–2.2 V	90 mAh g^{-1} (1.1 A g^{-1})	77%	70 mAh g^{-1} (100 cycles, 1.1 A g^{-1})	–	–	–	based on anode	3
3	NVP/C	1M $NaClO_4$ in PC	1 V–2.2 V	59.4 mAh g^{-1} (0.1 A g^{-1})	77%	54.28 mAh g^{-1} (100 cycles, 0.1 A g^{-1})	–	1.33	1	based on cathode	4
4	NVP/C nanosheet	1 M $NaClO_4$ in EC/DMC (1:1) + 5% FEC	1 V–2.4 V	85 mAh g^{-1} (1 C)	–	81 mAh g^{-1} (100 cycles, 1 C)	–	–	–	–	5
5	$Na_2LiV_2(PO_4)_3$/C	1 M $NaClO_4$ in EC/DEC	1 V–2.2 V	74.1 mAh g^{-1} (0.5 C)		47.8 mAh g^{-1} (200 cycles, 3 C)	37.2 mAh g^{-1} (1.2 A g^{-1})			based on anode	6
6	N, B codoped carbon coated 3D flower	1 M $NaClO_4$ in PC + 5% FEC	0.8 V–2.3 V	104 mAh g^{-1} (1.1 A g^{-1})	–	87 mAh g^{-1} (100 cycles, 1.1 A g^{-1})	–	2	1	based on cathode	7
7	NVP with in-situ carbon layers	1 M $NaPF_6$ in EC/DEC + 2% FEC	1 V–2.2 V	101.8 mAh g^{-1} (27.5 mA g^{-1})	–	70 mAh g^{-1} (200 cycles, 220 mA g^{-1})	52 mAh g^{-1} (1.1 A g^{-1})	–	–	based on anode	8
8	NVPC@CNFs	Na [FSA]-[C2C1im][FSA]	1.0 V–2.4 V	90 mAh g^{-1} (2 C)	–	54 mAh g^{-1} (3000 cycles, 2 C)	48.3 mAh g^{-1} (100 C)	2	1	–	9

9	NVP	0.9 M $NaClO_4$/TEP electrolyte	1.0 V−2.0 V	50.9 mAh g^{-1} (23.6 mAh g^{-1})	80.3%	44.2 mAh g^{-1} (500 cycles, 118 mA g^{-1})	35.1 mAh g^{-1} (1.88 A g^{-1})	1	0.8	based on anode	10
10	NVP/expanded graphite	1 M $NaClO_4$ in 95% PC + 5% FEC	1.0 V−2.0 V	92.2 mAh g^{-1} (1 C)	96.3%	80.9 mAh g^{-1} (100 cycles, 1 C)	43.8 mAh g^{-1} (10 C)	2.5	1	based on cathode	11
11	$Na_3V_{2-x}Ca_x(PO_4)_3$@C	1 M $NaClO_4$ in EC/DMC + 5% FEC	1.0 V–4.0 V	116.9 mAh g^{-1} (0.5 C)	–	75 mAh g^{-1} (2000 cycles, 10 C)	102 mAh g^{-1} (50 C)	1	1.5	–	12
12	$Na_3V_2(PO_4)_3$ with sulfur-doped carbon	1 M $NaClO_4$ in EC/DMC (1:1)	1.2 V–2.2 V	67.9 mAh g^{-1} (5 C)	–	68 mAh g^{-1} (1000 cycles, 5 C)	62 mAh g^{-1} (10 C)	–	–	–	13
13	3-D interconnected porous NVP	1 M $NaClO_4$ in PC + 5% FEC	1.0 V–2.1 V	73 mAh g^{-1} (1 C)	80%	28.9 mAh g^{-1} (500 cycles, 5 C)	47.7 mAh g^{-1} (5 C)	2	1	based on cathode	14
14	NVP on carbon cloth	1 M $NaClO_4$ in EC/DMC (1:1)	1.2 V–2.2 V	88.9 mAh g^{-1} (10 C)	–	78.3 mAh g^{-1} (2500 cycles, 10 C)	63 mAh g^{-1} (20 C)	–	–	–	15
15	K & Mg co-doped NVP	1 M $NaClO_4$ in PC	1.2 V–2.2 V		–	49 mAh g^{-1} (500 cycles, 10 C)	52.1 mAh g^{-1} (10 C)	2	1	based on cathode	16
16	Our NVP cell	1 M $NaPF_6$ in DME	0.5 V–2.3 V	99 mAh g^{-1} (0.1 A g^{-1})	95%	54 mAh g^{-1} (4000 cycles, 2 A g^{-1})	20 mAh g^{-1} (50 A g^{-1})	1	2.5	based on cathode	This report

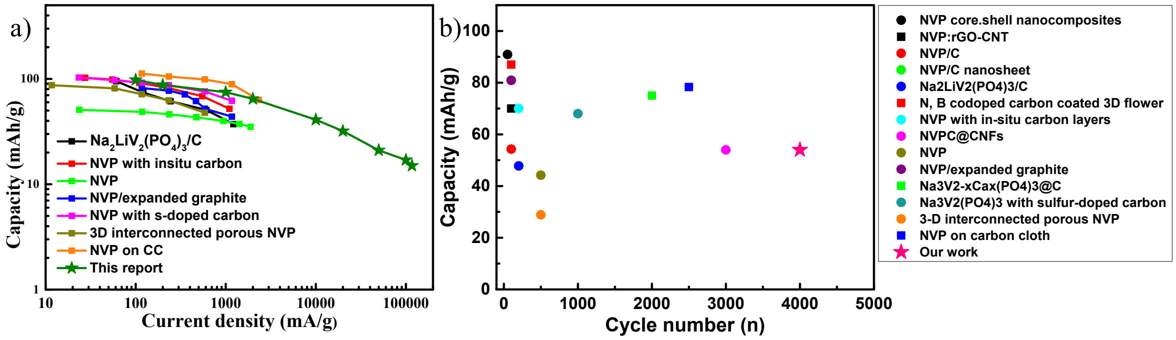

Figure S9. a) Comparison of our NVP cell's a) rate performance, and b) cycling performance with those of devices from previous reports (circle denotes a capacity less than 5 C, square denotes a capacity from 5 C–10 C, star denotes a capacity more than 10 C).

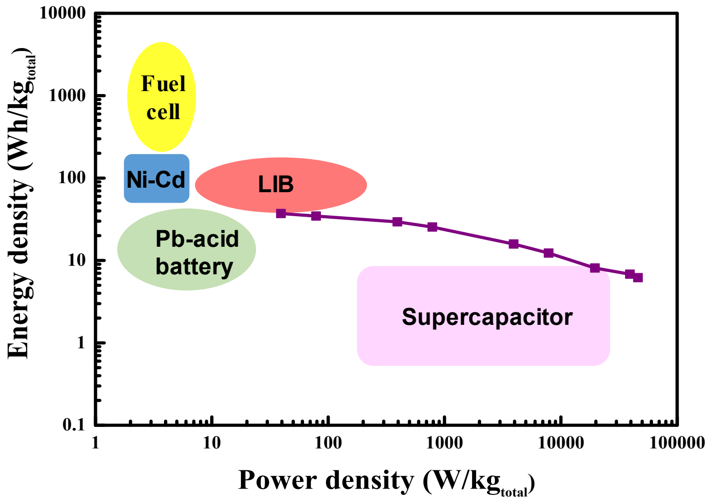

Figure S10. Ragone plot comparison of the symmetric NVP cell with present energy storage devices.

References

1. Sadan, M.K., Kim, H., Kim, C., Cha, S.H., Cho, K.-K., Kim, K.-W., Ahn, J.-H., adn Ahn, H.-J., *J. Mater. Chem. A,* 2020, **8***,* 9843.

2. Duan, W., Zhu, Z., Li, H., Hu, Z., Zhang, K., Cheng, F., and Chen, J., *J. Mater. Chem. A,* 2014, **2**, 8668.

3. Zhu, C., Kopold, P., van Aken, P.A., Maier, J., and Yu, Y., *Adv Mater.,* 2016**, 28**, 2409.

4. Hung, T.F., Cheng, W.J., Chang, W.S., Yang, C.C., Shen, C.C., Kuo, Y.L., *Chemistry* 2016**, 22**, 10620.

5. Ruan, Y., Liu, J., Song, S., Jiang, N., Battaglia, V., *Ionics,* 2017, **24**, 1663.

6. Wang, W., Xu, Q., Liu, H., Wang, Y., Xia, Y., *J. Mater. Chem. A,* 2017, **5**, 8440.

7. Jiang, Y., Zhou, X., Li, D., Cheng, X., Liu, F., Yu, Y., *Adv. Energy Mater.,* 2018, ***8***, 1800068.

8. Ling, R., Cai, S., Xie, D., Li, X., Wang, M., Lin, Y., Jiang, S., Shen, K., Xiong, K., Sun, X., *Chem. Eng.,* 2018**, 353**, 264.

9. Hwang, J., Matsumoto, K., Hagiwara, R., *ACS Appl. Energy Mater.,* 2019*,* ***2***, 2818.

10. Liu, X., Jiang, X., Zhong, F., Feng, X., Chen, W., Ai, X., Yang, H., Cao, Y., *ACS Appl. Mater. Interfaces*, 2019, **11**, 27833.

11. Gu, E., Xu, J., Du, Y., Ge, X., Zhu, X., Bao, J., Zhou, X., *J. Alloys Compd.* 2019, **788**, 240.

12. Zhao, L., Zhao, H., Du, Z., Wang, J., Long, X., Li, Z., Świerczek, K., *J. Mater. Chem. A,* 2019, *7*, 9807.

13. Li, W., Yao, Z., Zhong, Y., Zhou, C.-a., Wang, X., Xia, X., Xie, D., Wu, J., Gu, C., Tu, J., *J. Mater. Chem. A,* 2019, *7*, 10231.

14. Didwal, P.N., Verma, R., Min, C.-W., Park, C.-J., *J. Power Sources,* 2019, ***413***, 1.

15. Li, W., Yao, Z., Zhou, C.A., Wang, X., Xia, X., Gu, C., Tu, J., Small, 2019, ***15,*** 1902432.

16. Das, A., Majumder, S.B., Roy Chaudhuri, A., *J. Power Sources,* 2020, ***461***, 228149.
